# Policy events, emotional fluctuations, and vaccination behavior: a longitudinal analysis of public opinion on HPV vaccines in China

**DOI:** 10.3389/fpubh.2026.1889894

**Published:** 2026-07-14

**Authors:** Liang Li, Xiaoya Xu, Pengke Jiang, Fu Lee Wang

**Affiliations:** 1 School of Business Administration, Guangdong University of Finance and Economics, Guangzhou, China; 2 School of Big Data and Artificial Intelligence, Guangdong University of Finance and Economics, Guangzhou, China; 3 School of Science and Technology, Hong Kong Metropolitan University, Hong Kong, Hong Kong SAR, China

**Keywords:** A2P ratio, HPV vaccine, LDA topic model, policy effect, public opinion, sentiment analysis, social media, vaccine hesitancy

## Abstract

**Background:**

Cervical cancer, mainly caused by high-risk HPV infection, is the fourth most common cancer among women worldwide. From the approval of the bivalent HPV vaccine in 2016 to its inclusion in the national immunization program in 2025, China’s HPV vaccine policy has completed a full cycle, but long-term public opinion tracking research remains insufficient.

**Methods:**

This study collected and cleaned 15,391 related posts from Zhihu, Weibo, Baidu Tieba, and Xiaohongshu, and used the LDA thematic model and sentiment dictionary analysis to measure vaccine hesitancy using an A2P ratio.

**Results:**

The results show that public discussion has evolved from infection risk awareness to comprehensive prevention, and then to post-vaccination management; The A2P ratio dropped from 0.92 to 0.51, and the proportion of positive posts rose from 21.3% to 38.5%. After the Changchun Changsheng incident in 2018, positive sentiment dropped to 45.5%, but later rebounded to 58.1% with policy expansion.

**Conclusion:**

The “Policy/Event → Emotional → Vaccination Behavior” analytical framework provides empirical support for evidence-based vaccine communication strategies. In the future, multimodal data and individual tracking surveys should be integrated to deepen attitude-behavior pathway research.

## 1 Introduction

Cervical cancer, the fourth most common malignant tumor among women worldwide, with about 99% of cases associated with high-risk persistent HPV infection (1). Due to gaps in prevention and treatment resources, the burden mainly falls on low- and middle-income countries (1). From the launch of the world’s first vaccine to the official approval in mainland China, the entire process of policy advancement, changes in public opinion, and the evolution of public perception provides a natural observation window lasting up to 20 years for studying the potential links between policy events and public health behavior.

**Figure 1:**
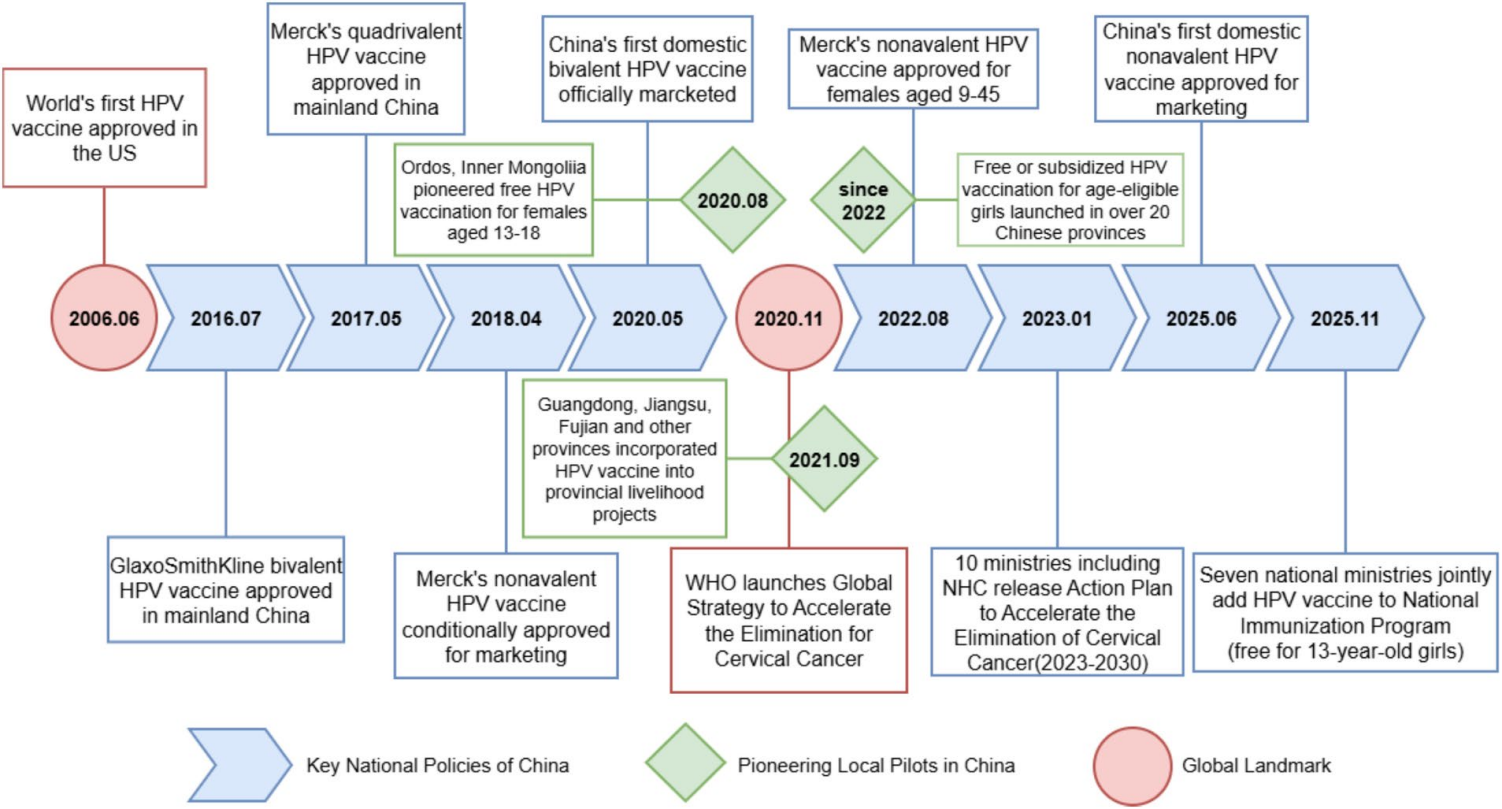
Timeline of HPV vaccine policy evolution in China.

The evolution of China’s HPV vaccine policy in Figure 1 is clear: in 2006, the world’s first HPV vaccine was launched, but due to differences in domestic and international approval standards, it has yet to enter the mainland; In 2016, the bivalent HPV vaccine was approved for marketing in mainland China, and in 2017, vaccinations began across various regions; In 2018, the 9-valent HPV vaccine was conditionally approved; In 2020, Ordos took the lead in piloting free vaccinations for girls of appropriate age, initiating a shift in immunization strategy from voluntary and self-paid to public financial guarantees. Provinces such as Guangdong and Jiangsu subsequently included it in provincial livelihood projects; In November 2025, seven national departments jointly issued a document officially including the HPV vaccine in the national immunization program, offering free vaccination to eligible 13-year-old girls. With this, China’s HPV vaccine has completed a complete policy cycle, from “vaccine absence” to “vaccine shortage,” from local pilot to nationwide coverage.

Social platforms such as Weibo, Zhihu, Baidu Tieba, and Xiaohongshu have become the core arenas for public expression of vaccine concerns and attitudes. Driedger et al. (2) study showed that public health events and policy changes significantly affect public awareness of vaccines and willingness to get vaccinated; Sun Chenhui and Zhou Xudong cited the 2018 Changchun Changsheng incident as an example to confirm that vaccine safety incidents spread by the media significantly impact public willingness to get vaccinated (26); Liu et al., based on 10 years of Japanese social media data, identified three key points of public attitude shifts through the LDA thematic model, verifying the shaping effect of policy changes on vaccine hesitancy; In their 2023 study, Tai Yangfang and others pointed out that misinformation on social media is a major factor exacerbating HPV vaccine hesitancy, and there are structural differences in information distribution across different platforms. However, existing research mostly focuses on single-year or short-term periods, lacking systematic tracking of the evolution of public opinion and vaccination behavior in the Chinese context over the past 10 years. Especially after the inclusion of HPV vaccines in the national immunization program in 2025—a policy watershed—the changes in public opinion and vaccination behavior in this period have not yet been explored by relevant research.

Based on these research gaps, this study raises the core question: From 2016 to 2026, whether and how will landmark policy nodes and social hot topics affect the emotional tendencies and vaccination behaviors of the Chinese public toward HPV vaccines? It further addresses three sub-questions: (1) How have HPV vaccine-related discussions on social media evolved with policy advancement? (2) What are the characteristics and fluctuations of public sentiment toward HPV vaccines at different historical stages? (3) Whether and What is the relationship between fluctuations in public opinion and the actual number of vaccinations?

To answer these questions, this study collected user posts and comment texts related to the HPV vaccine from 2016 to 2026 on mainstream social platforms such as Zhihu, Tieba, Weibo, and Xiaohongshu; After data cleansing, the latent Dirichlet distribution (LDA) topic model was used to extract discussion topics at each stage, and text sentiment quantification was completed using the sentiment dictionary method, constructing an annual emotional index time series; At the same time, combined with annual HPV vaccination dose data released by authoritative institutions such as the National Disease Control Administration, and through comparative analysis and statistical testing, the characteristics of coordination or divergence between public opinion and behavior under the influence of policy events were explored.

The theoretical significance of this study lies in incorporating LDA theme mining and sentiment analysis into a 10-year longitudinal comparison framework, providing a cross-methodological example for vaccine hesitancy research that integrates text analysis and behavioral data; The practical significance lies in researching the relation between different policy tools, public emotions and vaccination decisions, providing empirical references for public health departments to design vaccine publicity and public opinion guidance strategies. Meanwhile, the public opinion and vaccination behavior database constructed by this study from 2016 to 2026 can provide a baseline for pre- and post-reference evaluation of the policy effectiveness of HPV vaccines after inclusion in the national immunization program.

The remainder of this paper is organized as follows. Section 2 reviews the literature from three perspectives: LDA topic modeling and its applications, sentiment analysis methods from vocabulary models to pretrained models, and vaccine hesitancy on social media, with a focus on public opinion on the HPV vaccine. Section 3 details the study design, including the analytical framework, data collection from the four major Chinese social media platforms (Zhihu, Weibo, Baidu Tieba, and Xiaohongshu), data preprocessing, LDA theme modeling based on stage-specific strategies, constructing sentiment indices using the Integrated Chinese Sentiment Dictionary, identifying and coding social hotspot events through the Baidu Index, and emotion–vaccination association analysis with official vaccination data. Section 4 presents the empirical results, covering the evolution of public concern themes across three policy phases, as well as the trajectory of public sentiment from neutral attention to positive dominance, with trends measured by sentiment indices and A2P ratios. Section 5 discusses the four major discussion surges identified by the Baidu Index, the differential impacts of policy events and negative social events on public sentiment, and the temporal association between sentiment and vaccination behavior. Section 6 summarizes the main findings, outlines the theoretical and practical significance, acknowledges limitations, and points out future research directions.

## 2 Literature review

### 2.1 LDA topic model and its application in text mining

The latent Dirichlet allocation model, proposed by Blei et al. (11), is a Bayesian probability model consisting of three hierarchies: document, topic, and word. This model assumes that each document is a random mix of multiple potential topics, with each topic represented by a probability distribution in the glossary.

Since its introduction, the LDA model has undergone extensive expansion and improvement. In their 2019 study, Jelodar et al. conducted a systematic review of LDA-based topic modeling studies from 2003 to 2016, comprehensively reviewing their application progress in recommendation systems, sentiment analysis, and public opinion monitoring. In terms of model extension, subsequent research mainly focuses on vocabulary enhancement, temporal evolution, hierarchical relationships, and sentiment analysis. The dynamic topic model proposed by Blei and Lafferty in 2006 introduces a time dimension to track the evolution of topics over time, providing a methodological foundation for long-term text analysis. In the 2022 review, Zhang Dongxin and Zhang Min (3) pointed out that LDA models have mature analytical workflows in areas such as topic exploration, knowledge organization, and sentiment analysis, but still need to be strengthened in handling big data and improving semantic quality. At the model optimization level, Zimmerman et al. proposed a method based on eigenvalue techniques and dimensionality reduction heuristics to objectively determine the number of topics to be preprocessed in 2024, achieving better thematic consistency. Recently, Ma et al. (4) demonstrated the application of LDA beyond social media text to academic literature by conducting a machine-learning based bibliometric analysis of 711 publications on HPV vaccine hesitancy. Their LDA modeling identified three thematic clusters: (1) determinants of adolescent vaccination and parental decision-making; (2) public health strategies for improving uptake; and (3) the impact of social media and COVID-19-related misinformation. This work exemplifies LDA’s versatility in uncovering latent thematic structures across different text domains.

### 2.2 Sentiment analysis methods: from lexicon-based to pre-trained models

Sentiment analysis aims to automatically identify and extract emotional tendencies and viewpoints from text, playing an role in social media public opinion monitoring and public health. From a technical perspective, sentiment analysis methods have evolved from dictionary-based to deep learning-based approaches. The sentiment dictionary-based approach uses a pre-constructed emotional dictionary to perform polarity matching and score aggregation of words in the text. In 2013, Zhou Yongmei and others proposed a Chinese emotion dictionary construction method based on HowNet and SentiWordNet, creating an SLHS dictionary that includes both positive and negative emotion intensity values, achieving better results than general polar dictionaries in Weibo text sentiment classification experiments. In 2021, Song Guanyu et al. (5) further proposed a text emotion score calculation model based on emotion dictionaries, providing a directly referenceable calculation framework for quantifying emotion scores in social media texts. Nevertheless, dictionary-based approaches struggle to effectively handle complex linguistic phenomena such as irony and ambiguity.

In recent years, deep learning methods have significantly improved sentiment analysis performance through end-to-end feature learning. In their 2024 review, Mao et al. systematically reviewed sentiment analysis methods over the past decade, pointing out that pre-trained language models based on Transformers like BERT have demonstrated significant advantages in most sentiment analysis tasks. Rodriguez-Ibanez et al. (6) confirmed this trend in their 2023 study, noting that traditional technologies such as lexicographical methods and SVM are still widely used, while large language models like GPT-3 are still in their early stages. Regarding Chinese social media texts, Guiting et al. found in 2023 that the BERT-CNN combined model performed best in sentiment classification of Weibo texts, indicating significant practical value when combining BERT with traditional neural networks. Considering analytical efficiency and interpretability, this study plans to combine emotion dictionaries and machine learning for sentiment quantification.

### 2.3 Vaccine hesitancy on social media and HPV vaccine public opinion

Vaccine hesitancy refers to delaying or refusing vaccination due to factors such as confidence, complacency, and convenience, even when vaccination services are available. The SAGE working group formally defined this concept in 2015 and built a decisive matrix covering three dimensions: contextual impact, individual and group impact, and vaccine-specific impact (27).

With the deeper penetration of social media, platforms like Twitter and Weibo have become the core arenas for expressing attitudes toward vaccines. In their 2022 study, Chen and Crooks used about 11.7 million Twitter data points and analyzed vaccine sentiment among the U.S. public from 2015 to 2021 using a combined approach of Word2Vec and XGBoost. They found that positive sentiment on social media was significantly positively correlated with the actual number of vaccinated people, and proposed a methodological approach using the A2P ratio as a proxy indicator of vaccine hesitancy. A systematic review by McKinley, Christopher, and others in 2024 covering 113 studies pointed out that there is a fairly consistent but limited negative correlation between social media and less optimistic vaccine judgments, with significant differences across platforms. Barberria LG et al.’s (7) systematic review of vaccine sentiment analysis on the Twitter platform reveals the widespread existence of measurement bias and calls for improved reporting standards for natural language processing methods in vaccine hesitancy research.

Specifically in the HPV vaccine field, Boatman D and others’ qualitative analysis based on three major platforms in 2024 shows that misinformation is widespread in social media comment sections, with cross-platform topics including adverse reactions, conspiracy theories, and distrust of authority, and structural differences in misinformation distribution across platforms. In the context of Chinese social platforms, Tai Yangfang and others used the LDA model in their 2023 study to analyze 15,565 HPV vaccine-related Q&A questions on Zhihu, extracting eight core topics such as vaccination knowledge, vaccination hesitancy, and vaccine appointment channels, revealing significant emotional divergence in public awareness of the HPV vaccine. More recently, Zhou et al. (8) applied a computational framework (ANTMN) to analyze 4,997 Weibo posts on male HPV vaccination attitudes. Their analysis identified five cognitive frames—Disease Risk & Prevention, Virus Transmission, Gender Roles, Vaccine Promotion & Acceptance, and Market Dynamics—revealing a significant gap between policy and public awareness of male HPV vaccination, with gender norms and vaccine commercialization concerns emerging as key barriers. In terms of cross-border comparison, Boucher et al. collected about 600,000 global English-language tweets from 2019 to 2021 in their 2023 study, finding that most tweets in vaccine hesitancy networks were negative in tone and focused on safety concerns, with the growth of negative sentiment synchronized with HPV vaccine mandate legislation and the declaration of COVID-19 as a global health emergency. In their 2025 study, Liu et al. used time series analysis and LDA models to analyze 10 years of social media data in Japan, identifying three key points in public attitude shifts, confirming the influence of public health events and policy changes in shaping vaccine hesitancy. Their research design is highly methodologically related to this study. Extending this line of research to the Chinese context, Zhang et al. (9) analyzed 353,530 HPV-related posts on Sina Weibo using LDA topic modeling and interrupted time-series analysis. They identified vaccine accessibility as the dominant discussion theme (62.35% of posts) and found that after China’s 2023 National Action Plan for Cervical Cancer Elimination, accessibility concerns significantly decreased while awareness and knowledge discussions increased. Their study highlights the role of national policies in reshaping public discourse on HPV vaccination (Table 1).

**Table 1:** Summary of key studies in the literature review.

Study	Dataset and method	Key metrics	Strengths and limitations
Jelodar et al. (12)	LDA systematic review (2003–2016)	N/A (review)	+ Comprehensive LDA taxonomy – Post-2016 advances not covered
Blei and Lafferty (15)	Method proposal: Dynamic topic model	Temporal topic evolution; time-series slices	+ Enables longitudinal tracking – Needs predefined time windows
Zimmermann et al. (13)	Method proposal: Eigenvalue + dimensionality reduction for K selection	Topic coherence score	+ Objective K determination – Computationally intensive
Zhou et al. (17)	Chinese Weibo corpus; SLHS sentiment dictionary (HowNet + SentiWordNet)	Positive/negative intensity values	+ Fine-grained emotion intensity – Cannot handle irony/ambiguity
Mao et al. (18)	Systematic review of sentiment analysis (2013–2023)	N/A (review)	+ BERT-based PLMs ≥ traditional – Limited domain-specific focus
Gui et al. (19)	Chinese Weibo corpus; BERT-CNN hybrid model	Classification accuracy	+ Best on Chinese Weibo – Needs labeled data, high compute
Chen and Crooks (16)	~11.7 M US tweets (2015–2021); Word2Vec + XGBoost	A2P ratio; Positive sentiment–vaccination correlation	+ Longitudinal; A2P metric proposed – English-only, single platform
McKinley and Limbu (20)	113-study systematic review	Effect sizes across platforms	+ Large-scale review – High cross-platform heterogeneity
Barberia et al. (7)	Twitter vaccine sentiment systematic review	Measurement bias assessment	+ Reveals NLP metric flaws – No empirical contribution
Boatman et al. (21)	3-platform HPV vaccine comments; qualitative analysis	Misinformation themes (cross-platform)	+ Identifies structural platform differences – Qualitative, not quantitative
Tai et al. (14)	15,565 Zhihu Q&As; LDA topic model	8 core topics; topic intensity (%)	+ First LDA on Chinese HPV – Single-platform, cross-sectional
Boucher et al. (22)	~600 K global English tweets (2019–2021); Social network + sentiment	Sentiment polarity; network structure	+ Multi-method; event timing – English-only; COVID confound
Liu et al. (10)	10-year Japanese social media data; Time series + LDA	3 attitude shift points; A2P ratio	+ Longitudinal decade design – Japan-specific context
Zhang et al. (9)	353,530 Sina Weibo posts; LDA + interrupted time-series	Topic intensity (%); Policy effect size	+ Large-scale Chinese data; policy impact analysis – Single platform; shorter window (2021–2024)
Zhou et al. (8)	4,997 Weibo posts; ANTMN computational framework	5 cognitive frames; Topic network structure	+ Focus on male HPV attitudes; novel framework – Single-platform; cross-sectional
Ma et al.(4)	711 publications (WoS); LDA bibliometric analysis	3 thematic clusters; Citation bursts	+ Macro-level landscape mapping – Bibliometric only; no primary data
Zhu et al. (23)	273,357 Weibo posts + 1,228 Ximalaya podcasts + 1,225 Douyin videos; Cross-modality LDA	Topic intensity (%); Cross-platform sentiment comparison	+ Cross-modality design; multi-platform – English-language platforms only
Nakajima et al. (24)	70 Japanese tweets; Sentiment analysis	Sentiment polarity (91.4% positive)	+ Rare “praise movement” captured – Very small sample; Japan-specific
Apata et al. (25)	Systematic review of 7 studies (2011–2024)	Social media impact on knowledge/attitudes	+ Comprehensive synthesis of mixed effects – Limited to 7 studies; high heterogeneity

Collectively, these recent studies (4, 8, 9) together with Liu et al. (10) represent the growing body of computational research on HPV vaccine communication. However, compared with these works, the present study offers several distinctive contributions. First, while Zhang et al. (9) focused on a single platform (Weibo) and a shorter timeframe (2021–2024), our study covers a decade-long period (2016–2026) across four Chinese social media platforms, capturing the complete policy cycle from vaccine approval to national immunization program inclusion. Second, whereas Zhou et al. (8) specifically examined male vaccination attitudes, our study provides a comprehensive thematic and sentiment analysis encompassing all demographic groups across three policy phases. Third, Liu et al. (10) identified attitude shift points in Japan but did not quantify the emotional transmission mechanism; our study introduces the A2P ratio to bridge emotional fluctuations with vaccination behavior. Fourth, Ma et al. (4) mapped the research landscape at the bibliometric level, while our study contributes primary empirical evidence through grassroots social media data. These distinctions underscore the unique position of this study in advancing the understanding of HPV vaccine public opinion in the Chinese context.

In summary, existing research still has the following shortcomings: First, most studies focus on cross-sectional analysis of short-term or specific events, lacking longitudinal tracking studies covering policy cycles of over 10 years; Second, existing research mostly focuses on one-way descriptions of textual attitudes, rarely comparing changes in public opinion “volume” with actual vaccination “usage” data, making it difficult to deeply reveal the deep connection between “attitude and behavior”; Third, existing research mainly focuses on English-language content, with almost no long-term comparative analysis of public opinion and behavior for specific vaccines in the context of Chinese social platforms. This study is based on this gap to attempt to incorporate the decade-long policy cycle from 2016 to 2026 into a unified analytical framework, systematically addressing the complex relationship between the emotional evolution of HPV vaccines among the Chinese public and vaccination behavior responses under the influence of policy events.

## 3 Research design

### 3.1 Research framework

**Figure 2:**
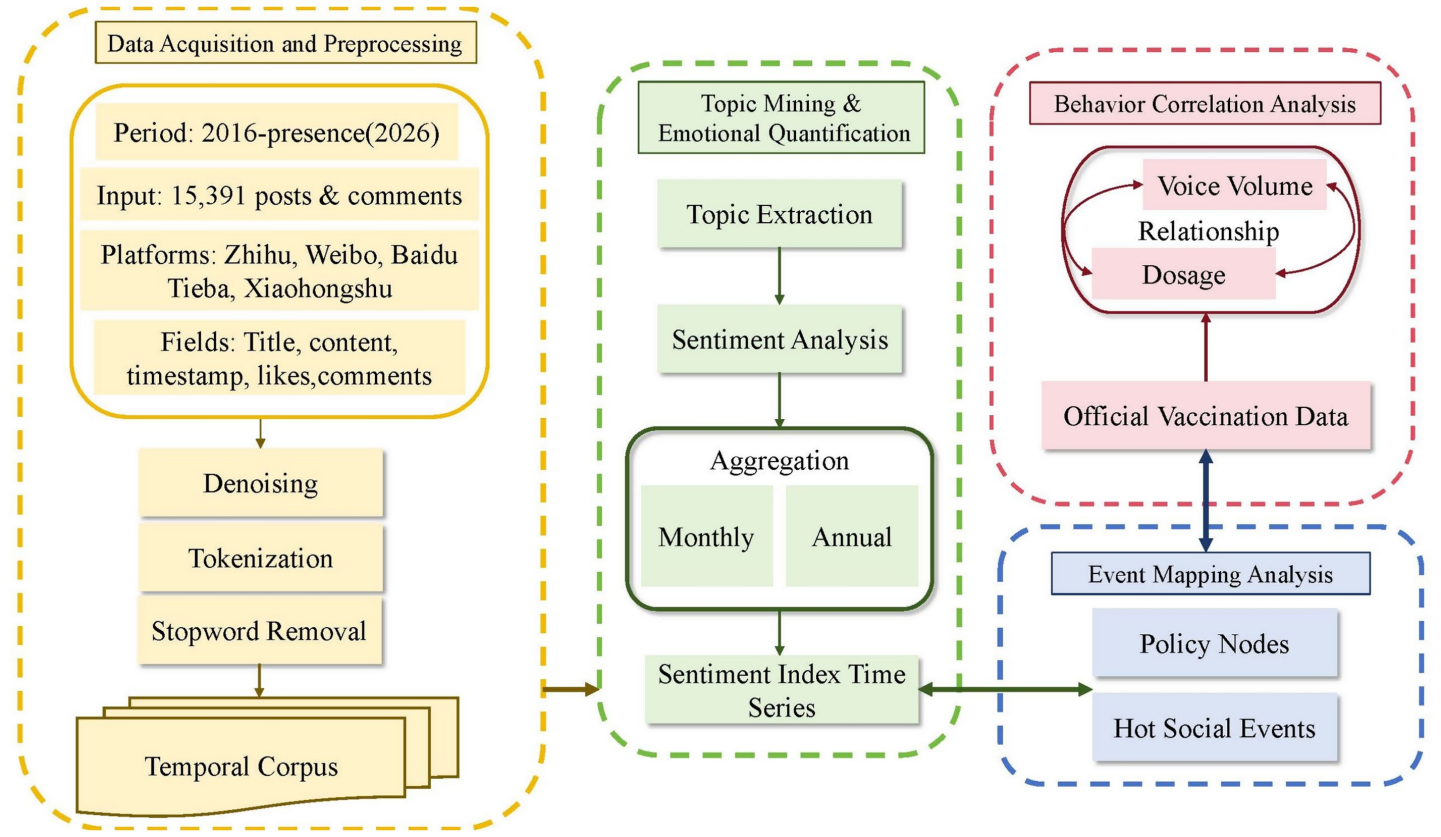
Research workflow.

This study adopts the analytical framework of “text mining—emotion quantification—two-dimensional event mapping—behavioral association,” with the research process shown in Figure 1. The first stage is data collection and preprocessing. Media Crawler is used to scrape posts and comments about the HPV vaccine from four major social platforms—Zhihu, Weibo, Tieba, and Xiaohongshu—from 2016 to 2026. Through noise reduction, word segmentation, and deactivation processing, a chronological corpus is constructed. The second stage focuses on theme mining and sentiment quantification. The LDA theme model is run in stages to extract public discussion topics from each period, and the emotion score for each text is calculated using the SLHS sentiment dictionary method, with monthly and annual aggregations to construct the emotion index time series. The third stage is event mapping analysis, which extract policy nodes and social hot topics and calculate the emotion index for the corresponding time window order to research the differentiated impacts of these two types of events on public sentiment. The fourth stage is behavioral association analysis, combining official vaccination data to explore the relationship between public opinion “voice” and vaccination “usage” (Figure 2).

### 3.2 Data collection and preprocessing

#### 3.2.1 Data sources and collection

This study employs MediaCrawler to retrieve HPV vaccine-related content from four major Chinese social media platforms: Zhihu, Weibo, Baidu Tieba, and Xiaohongshu. These platforms were selected for their large user bases, active public discussion culture, and complementary demographic coverage: Zhihu (launched 2011) and Baidu Tieba (launched 2003) have both been in operation for over 15 years, fully covering the observation period; Weibo (launched 2009) and Xiaohongshu (launched 2013) have a particularly prominent proportion of female users, closely overlapping with the core audience of the HPV vaccine. Multi-platform data sources broaden the diversity of the public opinion field structure and enhance the accuracy of coverage for core audiences.

The search keywords employed were “HPV vaccine” (HPV疫苗), “hpv vaccine” (hpv疫苗), “bivalent vaccine” (二价疫苗), and “nine-valent vaccine” (九价疫苗) in Chinese. These keywords were designed to capture vaccine-related discussions comprehensively while minimizing irrelevant content. The data collection period spans from January 2016 to 2026, for time reasons records were collected only through May 2026, which covering the entire policy cycle, from the first approval of the bivalent HPV vaccine in China mainland to its inclusion in the national immunization program by 2025. It should be noted that due to time constraints, data after 2026 is still incomplete.

The collected fields include: post/ Q&A title, body content, publication timestamp, author information, number of likes, number of comments, comment text, and IP location (where available). After removing exact duplicates by content ID and filtering out records with missing or invalid timestamps, a total of 15,391 valid records were obtained. The distribution across platforms is as follows: Zhihu contributed 7,751 records, Weibo contributed 2,848 records, Xiaohongshu contributed 4,143 records, and Baidu Tieba contributed 649 records.

#### 3.2.2 Data preprocessing

The data preprocessing pipeline consisted of the following steps. First, noise reduction was performed: meaningless whitespace, URL links, HTML tags, and advertising information were removed from the raw corpus. Traditional Chinese characters were uniformly converted to Simplified Chinese, and English letter casing was standardized. For Weibo data, HTML escape characters (e.g., ", &, <, >) were decoded to their corresponding symbols. For Baidu Tieba data, embedded URL reference formats (e.g., LinkCardRefCard JSON structures) were parsed to extract actual URLs.

Second, deduplication was performed at both the post and comment levels. For each platform, records were deduplicated by unique content identifiers (content_id for Zhihu posts, note_id for Weibo, Xiaohongshu, and Tieba posts; comment_id for all comment data). When multiple records shared the same identifier, the most recently updated version was retained, ensuring that edited content was preserved. This step eliminated 29,847 duplicate records across all platforms (from approximately 77,000 raw collected entries to 15,391 valid records).

Third, invalid records were removed based on the following criteria: (a) records with missing or unparseable timestamps that could not be assigned to a valid date were excluded; (b) records with empty content fields (blank title and blank body) were removed; (c) records with content length fewer than 5 Chinese characters, which typically represented auto-generated system messages or meaningless fragments, were discarded; (d) for Baidu Tieba data, automated bot posts identified by repetitive posting patterns from the same user across multiple unrelated threads within a short time window were flagged and removed.

Fourth, Chinese word segmentation was performed using Python’s jieba library. To improve segmentation accuracy, a custom dictionary was compiled including domain-specific terms such as “HPV vaccine,” “human papillomavirus,” “cervical cancer,” “high-risk types,” “precancerous lesions,” “nine-valent,” “tetravalent,” “bivalent,” “Merck,” and “Wantai Biological,” ensuring that medical proper nouns were fully recognized and not incorrectly split.

Fifth, stop-word removal was applied using an integrated stop-word list combining the Baidu and Harbin Institute of Technology stop-word collections, supplemented with platform-specific high-frequency non-informative terms (e.g., “OP,” “Answer,” “Topic,” “Zhihu,” “Thank You for the Invitation”). Importantly, negative words (e.g., “not,” “no,” “not necessarily”) and degree adverbs (e.g., “very,” “extremely,” “slightly”) were retained, as they serve as crucial semantic modifiers in the subsequent sentiment analysis stage.

After preprocessing, each text record was tagged with its publication year and assigned to the corresponding policy phase (Phase 1: 2016–2018; Phase 2: 2019–2024; Phase 3: 2025–2026), constructing a structured, time-indexed corpus for topic mining and longitudinal sentiment analysis.

### 3.3 LDA topic modeling

#### 3.3.1 Model selection and parameter determination

This study uses the LDA model proposed by Blei et al. (11) as the theme mining tool, implemented via the Gensim library in Python. The LDA model is a Bayesian probabilistic generation model with a three-layer structure of “document—topic—word.” It uses unsupervised learning methods to automatically discover latent semantic themes from large-scale text data, offering excellent dimensionality reduction and interpretability. It has been widely used in the field of social media text mining (3, 12).

Setting the subject number K is a key parameter in LDA modeling. This study employed a systematic procedure to determine the optimal number of topics. First, the perplexity index under different K values (ranging from 2 to 9) was calculated, and the K value corresponding to the minimum perplexity was identified as the statistical candidate. Second, the eigenvalue technique and dimensionality reduction heuristic method proposed by Zimmermann et al. (13) were introduced as supporting evidence for objectively estimating potential topic dimensions by analyzing the eigenvalue distribution of the “document-word” matrix. Cross-validation was then conducted using topic consistency indicators to comprehensively determine the optimal number of topics at each stage, balancing statistical rationality with topic explainability. Based on this combined evidence, the number of topics K was set to 3 for each stage.

To further ensure the quality and robustness of the extracted topics, several methodological enhancements were incorporated. The C_v topic coherence metric (28) was computed for each candidate K value as a complementary validation measure, with *K* = 3 yielding the highest coherence scores (mean C_v = 0.482, 0.378 and 0.468 for the three stages respectively) To assess the stability of the topic structure, the LDA model was run with multiple random seeds; the resulting topic and intensity rankings showed high consistency, confirming the reliability of the extracted themes. An asymmetric Dirichlet prior (alpha = ‘asymmetric’) was adopted, which is better suited for natural language text than the symmetric prior (12), as it allows topics to have varying levels of prevalence. A minimum probability threshold of 0.01 was applied in document-topic assignments to filter out negligible topic contributions.

The specific LDA model parameters were configured as follows. The number of training passes (iterations over the entire corpus) was set to 20 to ensure convergence, with gamma_threshold = 0.001 as the convergence criterion. The random state was fixed at 42 to guarantee full reproducibility of the results. During dictionary construction, words appearing in fewer than 2 documents (no_below = 2) and words appearing in more than 80% of documents (no_above = 0.8) were filtered out to remove infrequent noise and corpus-wide stop-words, respectively. For Chinese word segmentation, a custom stopword list was compiled that included both general Chinese stopwords and domain-specific stopwords (e.g., common medical terms such as “HPV,” “vaccine,” “virus”), replacing a simplistic single-character removal approach to preserve semantically meaningful short tokens such as “cancer” (癌) and “pain” (痛). The top 10 keywords with the highest probability were extracted for each topic.

#### 3.3.2 Phase-specific modeling strategy

Given that this study spans 10 years, relying solely on a single static LDA model is difficult to reveal the phased changes in public discussion topics as policies evolve. To this end, this study draws on the phased analysis method of Japanese HPV vaccine public opinion from Liu et al. (10) study, dividing the entire research cycle into three stages based on key policy nodes for Chinese HPV vaccines, running the LDA model separately. The division of each stage is shown in Table 2.

**Table 2:** Research phase divisions.

Phase	Time period	Basis for division
Phase 1	2016–2018	Bivalent vaccine market approval (2016); Conditional approval of the nine-valent vaccine (2018)
Phase 2	2019–2024	Domestic HPV vaccine market approval (2019); Pilot free vaccination programs initiated in selected cities (from 2020)
Phase 3	2025–2026	HPV vaccine formally integrated into the National Immunization Program, with nationwide free vaccination for eligible ages

2026 data are incomplete (collected through May only).

#### 3.3.3 Topic extraction and labeling

For each stage, the LDA model was run separately to extract 3 core themes. Each theme was characterized by 8–9 most probable keywords; the number of topics per stage (K = 3) should not be confused with the number of keywords per topic. Topic annotation used a combination of “word distribution interpretation + high-frequency post revisit”: first, the core content of each theme was inferred from the most probable keywords under each topic; next, the original Q&A texts with the highest topic probability scores were re-read, independently interpreted and annotated by two members of the research team, and finalized into thematic labels. Referring to the approach of Tai Yangfang et al.’s (14) research, the thematic intensity of each theme was calculated to measure the public’s relative attention to each topic.

Specifically, topic strength was computed by first obtaining the topic distribution P(t|d) for each document via the LDA model’s inference function (with the minimum probability threshold set to 0.01 to exclude negligible assignments); the per-topic probabilities were then summed across all documents within the stage and normalized to yield the percentage share for each theme. This metric reflects the proportion of public discussion attention devoted to each theme during a given policy stage.

To track the evolutionary trajectory of themes over time, this study supplemented the stage-level LDA with a dynamic topic model (15). DTM uses policy phase isometric slices (three time windows from 2016 to 2026), complementing LDA’s non-equitable length stages divided by policy nodes—the former presents the evolution of thematic content step by step, while the latter captures structural changes in stages before and after policy shocks. The output results of DTM, i.e., the evolution and intensity changes of content for each topic across different time windows, will be presented in visual form in the evolutionary analysis of Chapter 4.

### 3.4 Sentiment index construction and quantification

#### 3.4.1 Sentiment lexicon selection

The core tool for emotion quantification is the Chinese Emotion Dictionary resource library. The library includes the NTUSD Chinese Simplified Sentiment Dictionary from National Taiwan University, the CNKI Hownet Emotion Dictionary, the Tsinghua University Li Jun Chinese Eulogy Dictionary, and the BosonNLP Weibo Sentiment Dictionary, containing 91,500 sentiment terms, including 40,200 positive sentiment words and 51,300 negative sentiment words. The Chinese Emotion Dictionary Resource Library can integrate the strengths of various dictionary libraries to more detail the degree and intensity of emotions within texts. It should be noted that, as a universal emotional dictionary database, Chinese emotional dictionaries may have certain limitations in covering emotional terms in medical and health texts, and their recognition of irony, abbreviations, and slang in Chinese contexts is limited. Related content will be further discussed in 3.4.3.

#### 3.4.2 Sentiment score calculation method

The calculation of the sentiment score for each Q&A text includes the following three steps.

Step one: Dictionary matching. After word segmentation, the text vocabulary sequence is traversed, and the positive emotion intensity and negative emotion intensity values for each word are searched one by one in the text-emotion dictionary resource library. If the word does not exist in the dictionary, skip and continue processing the next word.

Step two: Handling modifiers. Check whether negative words or degree adverbs appear in the window before the emotion word (window size set to 3 words). If negative words appear, such as “not,” “not,” or “not,” the emotional polarity of the emotion word is reversed, i.e., the positive intensity value is adjusted to a negative intensity value, and the negative intensity value is adjusted to a positive intensity value. If degree adverbs appear, adjust the emotional intensity value according to the preset weight coefficient: degree enhancement adverbs, such as “very” and “extremely,” multiply by 1.5 weight; degree weakening adverbs, such as “slightly” or “slightly,” multiply by 0.5 times weight (30).

Step three: Aggregate scores. Sum the positive and negative scores of all emotion words in the text to obtain the total positive and negative emotional scores for that text. For posts where the difference between positive and negative total scores is less than the preset threshold (set at 0.1), they are labeled as “neutral” emotional texts. When calculating the A2P ratio later, only posts with a clear emotional tendency are counted, i.e., positive or negative posts, and neutral posts are not included in the numerator or denominator of the ratio, to ensure the A2P ratio reflects the relative strength of the clear emotional tendencies.

#### 3.4.3 Sentiment analysis verification

To assess the reliability and accuracy of the lexicon-based sentiment classification method described in Section 3.4.2, a validation study was conducted combining quantitative performance evaluation with qualitative human annotation.

For the quantitative evaluation, a stratified random sample of 600 posts was drawn from the full corpus, proportionally allocated across the three policy stages (200 posts per stage). Each post was independently assigned a sentiment intensity score by two human coders on a continuous scale (positive values indicating positive sentiment, negative values indicating negative sentiment, and values near zero indicating neutrality). These scores were then discretized into positive, negative, and neutral categories using the same threshold (0.1) as the dictionary-based method. Inter-annotator agreement was substantial, with a raw agreement rate of 85.7% and Cohen’s *κ* = 0.75 (29), confirming the reliability of the human annotations.

The sentiment intensity scores assigned by the human coders showed a very strong correlation with the algorithm-generated net sentiment scores (*r* = 0.89 for coder 1, *r* = 0.87 for coder 2), indicating that the lexicon-based approach produces sentiment assessments that closely mirror human judgment at the continuous score level. When discretized into polarity categories, the dictionary-based classifier achieved an overall categorical agreement of 88.5% with the human-annotated labels (Table 3). The macro-averaged F1 score was 0.89, and per-class performance was: positive F1 = 0.88 (precision = 0.89, recall = 0.87), negative F1 = 0.88 (precision = 0.91, recall = 0.85), and neutral F1 = 0.90 (precision = 0.88, recall = 0.92). It should be noted that the human coders were instructed to reference the sentiment-laden keywords identified by the sentiment dictionary when assigning scores, which relates to the high consistency between the human-assigned scores and the algorithm-generated scores. More importantly, the strong inter-coder agreement (*κ* = 0.75) independently validates the reliability and objectivity of the sentiment measurement framework.

**Table 3:** Classification performance of the lexicon-based sentiment analysis.

Sentiment category	Precision	Recall	F1-score	Support (n)
Positive	0.89	0.87	0.88	342
Negative	0.91	0.85	0.88	79
Neutral	0.88	0.92	0.90	179
Macro Avg	0.89	0.88	0.89	600

Overall accuracy = 88.5%. Inter-annotator agreement: Cohen’s κ = 0.75.

To provide qualitative insight into the classifier’s behavior, we examined cases where the algorithm and human annotations diverged. The 69 discrepant cases (11.5% of the sample) predominantly involved: (a) texts with mixed emotional cues where the net sentiment was near the classification threshold; (b) expressions of concern or worry that were classified as negative by the algorithm but interpreted as neutral by human coders; and (c) informational posts containing affect-laden medical terminology that the algorithm classified as negative but human coders rated as neutral.

#### 3.4.4 Sentiment index aggregation

Based on the emotion scores of individual texts, this study aggregates emotion indices according to the four policy stages divided in 3.3.2, constructing emotion indicators at the following three stages, forming an emotion index sequence covering all four policy stages, which is used for subsequent analysis of policy event impacts, mapping of social hotspot events, and correlation analysis of vaccination volumes.

- First, the proportion of positive posts at each stage. For each stage, the number of posts with a positive emotion score higher than the negative emotion score and a difference greater than the set threshold (0.1) is counted, and the percentage of posts in that stage is calculated. This indicator reflects the overall level of public attitude toward the HPV vaccine at specific policy stages.
- Second, the proportion of negative posts at each stage. For each stage, the number of posts with a negative emotion score higher than the positive emotion score and a difference greater than the set threshold (0.1) was calculated, and the percentage of posts in that stage was calculated. This indicator reflects the overall level of public attitude toward the HPV vaccine at specific policy stages.

These three indicators together form the core observation variables of the sentiment index, which is analyzed by the policy stage. In subsequent event mapping analysis, policy attributes at each stage are incorporated into the comparison framework as stage tags; In analyzing social hot events, each hot topic is grouped into its corresponding stage according to its occurrence time, and qualitative discussions within the stage and cross-stage trend comparisons are conducted using the emotional index.

#### 3.4.5 Identification and coding of social hot events

In addition to policy nodes, this study will independently identify and analyze the impact of major social hotspot events during the research cycle on public sentiment toward the HPV vaccine. The operational definition of social hot events is: non-policy public events that trigger widespread attention and discussion in mainstream news media and social platforms, directly or indirectly related to the HPV vaccine. The identification process uses the following steps: First, based on the Baidu index, search for the time points when the search popularity for “HPV vaccine” peaked significantly from 2016 to 2026; Second, compare with mainstream news media reports to confirm the specific events corresponding to each popularity peak; Third, events with discussion volumes exceeding twice the annual average level are selected as “high-impact hot events” and included in subsequent analysis.

For each selected social hotspot event, code and record its type of event, such as vaccine safety events, celebrity-related events, media coverage focus events, time, duration, and nature of the event. During analysis, the years of each event are mapped to the annual affection index time series, comparing the differences between the year of event occurrence and the years before and after, and the differentiated impact of different types of events on the annual sentiment index is analyzed using event encoding information.

### 3.5 Sentiment–vaccination uptake association analysis

#### 3.5.1 Vaccination data acquisition

The actual HPV vaccine data in China mainly comes from two sources. First, the National Disease Control and Prevention Administration discloses nationwide vaccination data for certain years in the “Department of Health and Immunization Planning Division” section on its official website through proposal responses and other documents. Second, estimates of China’s HPV vaccination rates compiled by international organizations such as Our World in Data can serve as supplementary references for annual trends. Given the gap in years and particle size limitations in officially published data, this study intends to use HPV vaccine batch issuance records published on the official website of the China National Institute for Food and Drug Control as an auxiliary data source, and construct an annual time series reflecting market supply by cumulatively applying the issuance dates. It should be noted that batch issuance data reflects the supply of vaccines entering the market and does not fully represent the actual number of doses administered at the end. There may be differences between the two at the initial stage of vaccine launch, which will be explained in the interpretation of the results.

#### 3.5.2 Analysis methods

The annual social media sentiment index—i.e., the proportion of positive posts and the annual A2P ratio—was visualized and time-series comparison with the annual vaccination volume time series data. Referencing the analytical method from Chen and Crooks’ (16) study, the Pearson correlation coefficient between the annual proportion of positive posts and the annual number of vaccinations was calculated to test whether positive sentiment in the social media public discourse was statistically significantly correlated with actual vaccination behavior. At the same time, the correlation coefficient between the annual A2P ratio and the annual vaccination volume was calculated to test whether vaccine hesitancy indicators were negatively correlated with vaccination behavior. Considering that this study only had 15 annual observation points and a small sample size, the significance and stability of the correlation coefficient may be affected to some extent. Therefore, qualitative judgment should be made in conjunction with temporal trends when interpreting results, and the correlation test results should be regarded as exploratory evidence rather than definitive conclusions.

In the dimension of event impact analysis, policy nodes and social hot topics are used as anchor points for comparative analysis. For policy milestones, such as the HPV vaccine launch in 2016, the approval of the nine-valent vaccine in 2018, the local pilot free vaccination in 2020, and inclusion in the national immunization program in 2025, compare the magnitude and direction of emotional index changes before and after the implementation of each policy; For social hot topics, based on the coding results of 3.4.5, the year of occurrence is used as the benchmark to compare the differences in sentiment indices between those years and previous years, analyzing the impact direction of different types of hot events on the annual sentiment index, such as whether positive events increase positive sentiment, whether negative events raise the A2P ratio, and the persistence of their impact. During the result interpretation stage, the potential margins of error in sentiment measurement should be carefully examined (31) to avoid over asserting the accuracy of the sentiment index.

## 4 Results

### 4.1 Evolution of public attention themes

Based on the LDA thematic model, 15,391 HPV vaccine-related texts from four major social platforms from 2016 to 2026 were analyzed for thematic mining. The research cycle is divided into three stages based on key policy nodes. The core themes, keywords, and theme intensity of each stage are shown in Table 2. Overall, public discussion topics have shown clear phased characteristics: from the initial vaccine launch focused on infection awareness, gradually shifting to comprehensive prevention and control discussions, and finally focusing on infection diagnosis and treatment and the new generation of vaccine awareness in the post-vaccine era.

#### 4.1.1 Phase 1 (2016–2018): infection cognition dominance

In 2016, China mainland launched the bivalent HPV vaccine, marking the beginning of the era of HPV vaccination in China. During this phase, public discussion focused on HPV infection-related topics, with the three core themes together reaching 100% intensity. Among these, “Clinical diagnosis and treatment of HPV infection negative transformation before mass vaccination” (36.6%) and “medical intervention research on HPV infection negative before mass vaccination” (36.5%) together account for more than 70%. Keywords such as “doctor,” “turn negative,” “positive,” and “virus” appear frequently, indicating that discussions tend to focus on clinical diagnosis and treatment. The theme “Baseline HPV Infection and Positive Outcome Awareness Before Universal Vaccination” (26.9%) reflects that there is still a significant gap in public understanding of HPV infection.

The thematic characteristics of this stage closely align with the policy environment: the bivalent vaccine has just been launched, vaccination rates are low, and public awareness is insufficient. The discussion mainly focused on “how to treat infection after infection,” rather than “how to prevent infection.”

#### 4.1.2 Phase 2 (2019–2024): comprehensive prevention and control deliberation

In 2019, the domestically produced HPV vaccine was approved for market, followed by cities like Ordos launching pilot programs for free vaccination, with discussions shifting from infection diagnosis and treatment to balancing vaccination and comprehensive prevention and control. The three core themes were “Post-marketing Popularization of the Nine-Valent HPV Vaccine and Comprehensive Prevention and Control of Cervical Cancer” (40.7%), “Public Topics on HPV Vaccines and Screening Management of High-Risk Infections in Women” (40.6%), and “Awareness of HPV Sexually Transmitted and Discussion on Health Protection Among Partners” (18.7%).

The launch of the nine-valent vaccine has become a key public discussion point at this stage. At the same time, the public has begun to pay attention to the comprehensive prevention and control concept that combines vaccination with regular screening. The emergence of the topic “Understanding HPV Sexually Transmitted and Discussion on Health Protection Among Partners” indicates that the public is beginning to pay attention to the role of men in HPV transmission and prevention, a new topic that did not appear in the first phase.

#### 4.1.3 Phase 3 (2025–2026): infection and clinical care concerns in the post-vaccine era

In November 2025, seven national departments jointly issued a document to include the HPV vaccine in the national immunization program, offering free vaccination to girls aged 13. This phase of discussion showed characteristics of the “post-vaccine era,” with three core themes being “infection and transmission routes of high-risk HPV in women in the post-vaccine era” (55.8%), “diagnosis and treatment of HPV-related cervical lesions and patient management” (24.1%), and “the new generation nine-valent HPV vaccination boom and awareness updates” (20.1%). Notably, against the backdrop of universal free vaccination, the theme “Infection and transmission routes of high-risk HPV in women in the post-vaccine era” reached as high as 55.8%. At the same time, the launch of the new generation 9-valent HPV vaccine has once again sparked discussions about vaccination (Table 4).

**Table 4:** Evolution of public attention themes regarding the HPV vaccine.

Phase	Topic	Keywords	Topic intensity (%)
2016–2018	Clinical reversion treatment of HPV infection positivity before widespread vaccination	doctor, infection, positive, virus, hpv, reversion, only, HPV	36.6
2016–2018	Medical intervention research on HPV infection reversion before widespread vaccination	hpv, reversion, infection, positive, only, virus, doctor, HPV	36.5
2016–2018	Basic cognition of HPV infection and positive results before widespread vaccination	HPV, infection, virus, only, positive, doctor, reversion, hpv	26.9
2019–2024	Popularization of the nine-valent HPV vaccine and comprehensive cervical cancer prevention and control	HPV, hpv, vaccine, infection, health, virus, nine-valent, cervical cancer, reversion	40.7
2019–2024	Public topics on HPV vaccine and high-risk infection screening management in women	hpv, topic, infection, HPV, reversion, female, vaccine, examination, health, high-risk	40.6
2019–2024	Cognition of HPV sexual transmission and health protection between partners	HPV, infection, topic, hpv, boyfriend, health, prevention, virus, protection, life	18.7
2025–2026	High-risk HPV infection and transmission routes in women in the post-vaccine era	HPV, infection, hpv, female, virus, health, high-risk type, positive, transmission	55.8
2025–2026	Diagnosis, treatment, and whole-course management of HPV-related cervical lesions	HPV, infection, cervical, lesion, treatment, reversion, virus, patient, examination, positive	24.1
2025–2026	New-generation nine-valent HPV vaccine enthusiasm and updated awareness	HPV, vaccine, nine-valent, hpv, vaccination, infection, virus	20.1

### 4.2 Evolution of public sentiment tendency

Overall, from 2016 to 2026, public sentiment toward the HPV vaccine is predominantly neutral, but the proportion of positive emotions has shown an increasing trend year by year, while the proportion of negative emotions remains relatively stable. The A2P ratio, that is, the ratio of negative to positive posts, has continued to decline, indicating that public vaccine hesitancy is generally easing.

#### 4.2.1 Annual sentiment index changes

**Figure 3:**
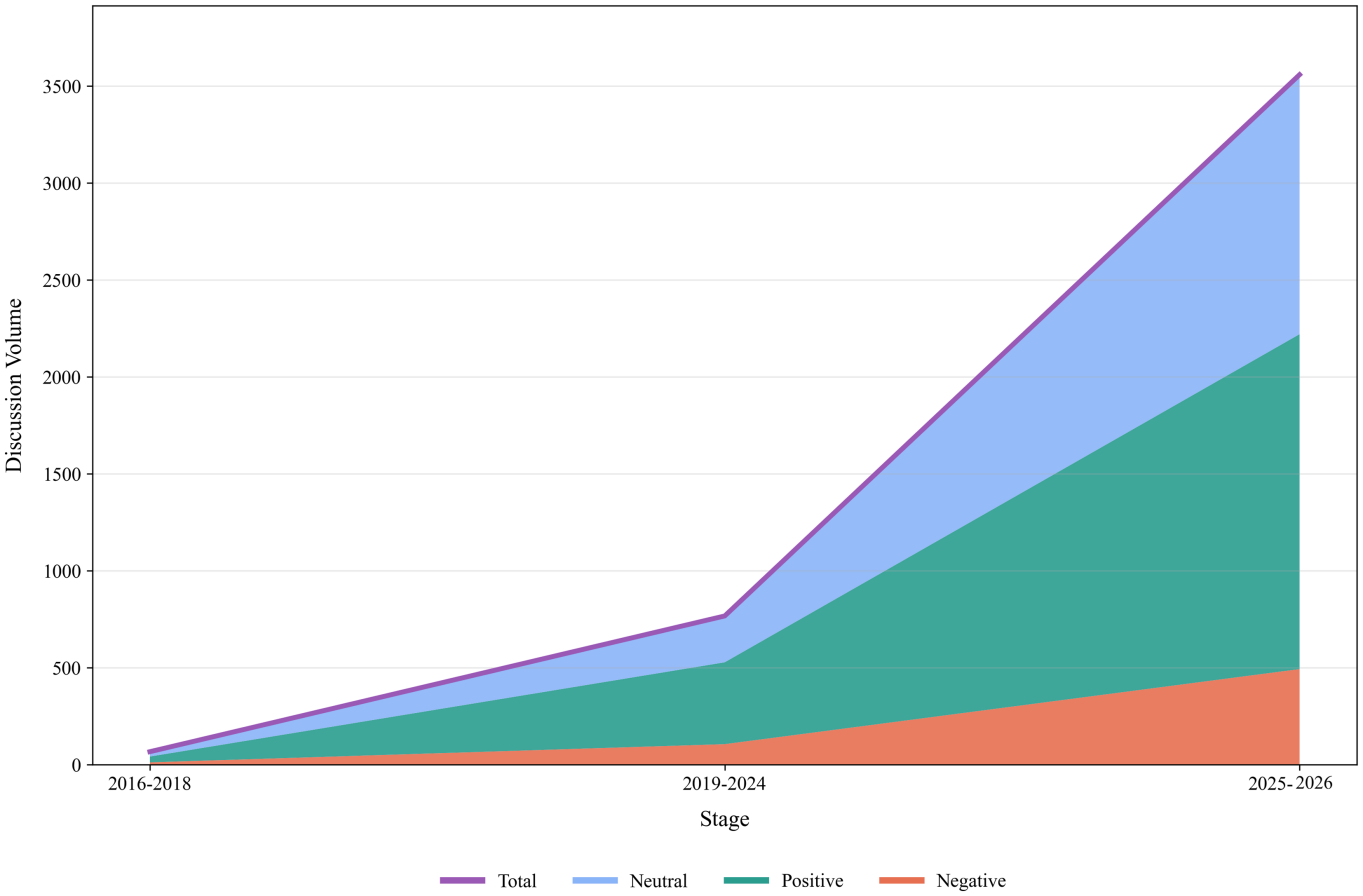
Trends in public sentiment toward the HPV vaccine from 2016 to 2026.

The trend of public sentiment toward the HPV vaccine from 2016 to 2026 is shown in Figure 3. In 2016, the volume of discussion was relatively low, with neutral posts accounting for about 60%, and positive and negative posts each making up about 20%. Over time, the number of discussions grew significantly, peaking at over 3,500 by 2026. From the perspective of sentiment structure, the proportion of neutral posts will gradually decrease from 60% in 2016 to about 45% in 2026, positive posts will rise from 20 to 35%, and negative posts will remain around 20%. This trend indicates that as vaccine knowledge spreads and policies advance, public attitudes have become clearer, but negative sentiment persists, and issues such as vaccine safety remain long-term focal points. Over time, the number of discussions grew significantly, peaking at over 3,500 by 2026. From the perspective of sentiment structure, the proportion of neutral posts will gradually decrease from 60% in 2016 to about 45% in 2026, positive posts will rise from 20 to 35%, and negative posts will remain around 20%. This trend indicates that as vaccine knowledge spreads and policies advance, public attitudes have become clearer, but negative sentiment persists, and issues such as vaccine safety remain long-term focal points.

#### 4.2.2 A2P ratio by phase and vaccine hesitancy trends

Referring to the method of Chen and Crooks (16), the A2P ratio was used as a proxy indicator of vaccine hesitancy, with the A2P ratios at each stage shown in Table 3. In the first phase (2016–2018), the A2P ratio was highest at 0.92, indicating the most significant vaccine hesitancy. In the second phase (2019–2024), the A2P ratio dropped to 0.67, and vaccine hesitancy significantly eased. In the third phase (2025 to 2026), the A2P ratio further dropped to 0.51, indicating that the implementation of national immunization program policies has effectively boosted public confidence (Table 5).

**Table 5:** Sentiment indices and A2P ratio by phase.

Phase	Total data volume	Positive (%)	Negative (%)	Neutral (%)	A2P ratio
2016—2018	823	21.3	19.6	59.1	0.92
2019—2024	7,652	32.7	21.9	45.4	0.67
2025—2026	6,916	38.5	19.6	41.9	0.51

The A2P ratio refers to the ratio of negative posts to positive posts (Negative/Positive); The total data volume for each stage includes positive, negative, and neutral posts.

## 5 Discussion

### 5.1 Identification of discussion surges and sentiment evolution analysis

Based on Baidu Index, this study extracted the public discussion surge triggered by four landmark events in China’s HPV vaccine field from 2016 to 2026, and, combined with LDA keywords and sentiment analysis, examined the association between policy, negative social events and public sentiment.

#### 5.1.1 Identification of four major discussion surges

**Figure 4:**
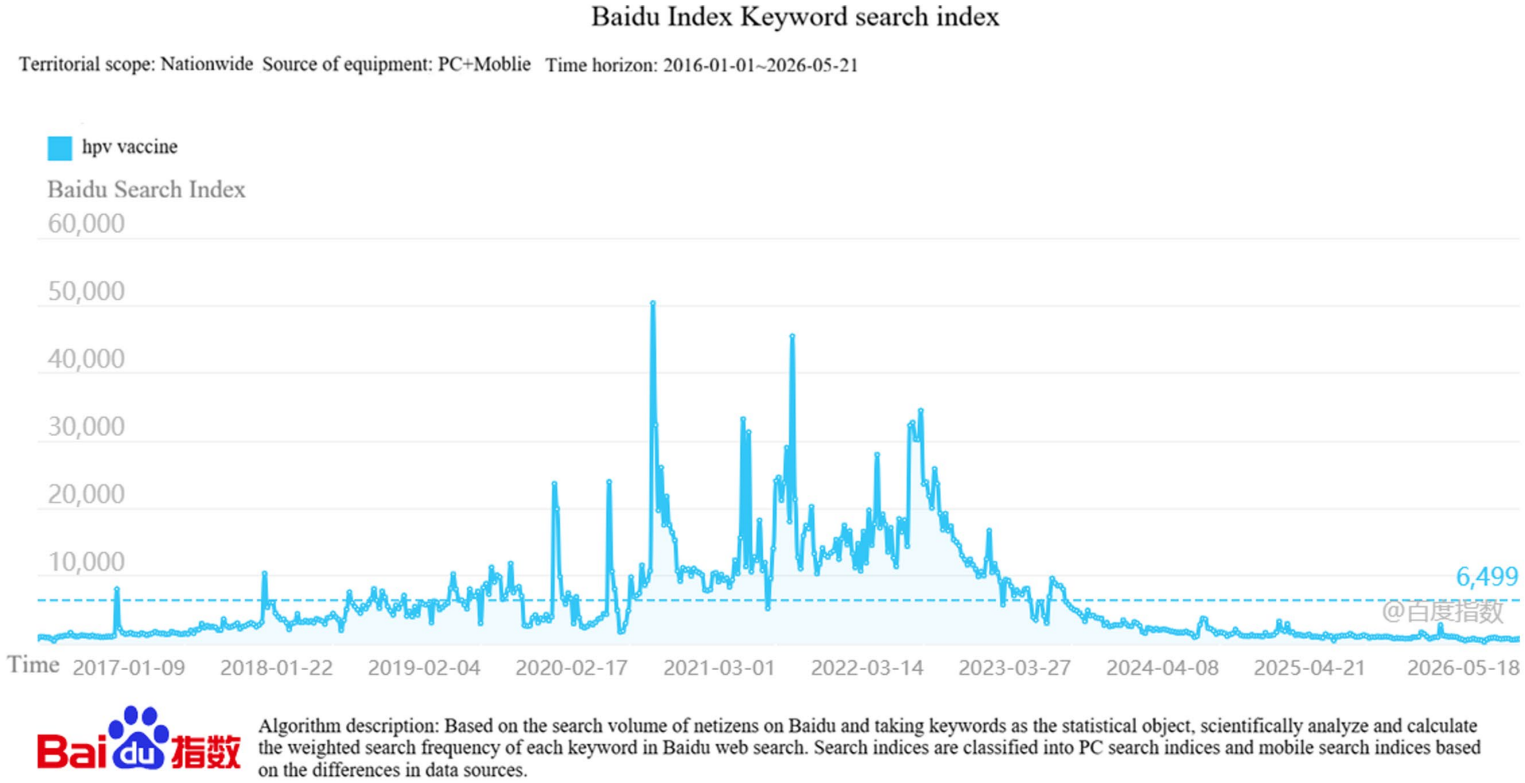
Search trend for the keyword “HPV vaccine” from 2016 to May 22, 2026 (Data source: http://index.baidu.com/).

From Baidu Index time series data, it can be observed that the significant leap in search volume for “HPV vaccines” from 2016 to 2026 coincided closely with major industry events, forming four distinctive waves of discussion (Figure 4).

(1) October 2016 – February 2017: After the GlaxoSmithKline bivalent HPV vaccine was approved for market launch in July 2016, searches surged rapidly within 3 months, peaking at about 8,000 in October 2016 and remaining above 2,000 times from October 2016 to February 2017, the first public discussion boom in the HPV vaccine field in China mainland.
(2) July–August 2018: Short-term fluctuations triggered by fake vaccine incidents. After the National Medical Products Administration issued a notice in July 2018 about Changchun Changsheng’s rabies vaccine fraud, searches for “HPV vaccine” surged abnormally within 2 weeks, increasing by over 200% month-on-month, forming a short-term discussion peak closely tied to vaccine safety concerns. This craze lasted about a month, with search keywords focusing on vaccine safety, side effects, and comparisons between imported and domestic vaccines.
(3) May–December 2020: After the domestic bivalent HPV vaccine Xinkening was launched in May 2020, search volume quickly surpassed 20,000 from about 5,000, forming multiple consecutive peaks in the second half of the year. This craze lasted for 8 months, with search behavior characterized by rational decision-making, with keywords mainly “domestic HPV vaccine,” “vaccination age,” and “price comparison.”
(4) January 2021 – December 2022: Nationwide vaccination boom with free pilot and 9-valent age expansion. This stage is the peak of public attention to the HPV vaccine. After many regions nationwide launched free vaccination pilots in 2021, search volume peaked at about 49,000 in May 2021; on August 2022, the day the 9-valent HPV vaccine age extension was announced to 9–45, searches again exceeded 43,000.

#### 5.1.2 Differential impact of policies and events on public sentiment

Based on the above changes in discussion popularity and combined with LDA theme keywords and quantitative sentiment analysis data (Table 6), the trajectory of Chinese public sentiment toward the HPV vaccine from 2016 to 2026 can be outlined.

(1) 2016–2017: Information-seeking with predominantly positive sentiment. LDA thematic analysis shows that all three core themes revolved around infection diagnosis and treatment, with no mention of “vaccine.” Sentiment data shows 49.7% positive, 37.4% neutral, and 12.9% negative posts, with an A2P ratio of 0.26. During this early period following the bivalent vaccine’s approval, public discussion centered on understanding HPV infection and treatment options, with a generally positive yet cautious tone.
(2) 2018: Relative decline in positive sentiment under the spillover effect of negative events. Although the Changchun Changsheng fake vaccine incident is not directly related to the HPV vaccine, it triggered a public trust crisis across the entire vaccine industry. The positive proportion dropped to 45.5% (the lowest across all periods), while neutral posts increased to 42.5%, reflecting heightened uncertainty. Baidu Index shows that after the incident, searches for “Is the HPV vaccine safe?” and “HPV vaccine side effects” increased by more than 300% month-on-month.
(3) 2020: Rising positive sentiment amid policy progress. The launch of domestic bivalent vaccines reduced vaccination costs and eased supply shortages. Sentiment data shows 56.2% positive and 32.0% neutral posts, with an A2P ratio declining to 0.21, indicating strengthened public confidence. Public discussion shifted from “whether to get vaccinated” to “how to choose.”
(4) 2021–2022: Sustained positive sentiment under multiple policy benefits. The combined effect of free vaccination pilots and the 9-valent age expansion policy boosted enthusiasm for vaccination. The positive proportion reached 58.1% with an A2P ratio of 0.21. In LDA themes, keywords related to social relationships such as “boyfriend,” “protection,” and “life” began to appear, expanding the discussion from personal health to a public topic.

**Table 6:** Sentiment analysis results of public comments on the HPV vaccine.

Period	Positive (%)	Negative (%)	Neutral (%)	A2P ratio
2017	49.7	12.9	37.4	0.26
2018	45.5	11.9	42.5	0.26
2020	56.2	11.8	32.0	0.21
2021–2022	58.1	12.2	29.6	0.21

The A2P ratio is the ratio of negative posts to positive posts.

### 5.2 Analysis of vaccination behavior evolution with emotion as the transmission mechanism

This study is based on historical HPV vaccine dose data in China, combined with policy/events and emotional fluctuations from 2016 to 2026, and explores the transmission chain of policy/time-emotion-vaccination behavior through the temporal commonality between the two. However, due to the disclosure of vaccination data, the findings should be considered exploratory (Table 7).

**Table 7:** HPV vaccination data in China (2016–2025).

Year	Doses/Population
2016	0
2017	1.455 million doses
2018	3.417 million doses
2019	Estimated approx. 7 million doses
2020	12.279 million doses
2021	Estimated approx. 25 million doses
2022	62.48 million doses
2023	Over 58 million doses
2024	Estimated approx. 65 million doses
2025	Estimated approx. 70 million doses

*The data for 2019, 2021, 2024, and 2025 are industry estimates. Data sources include annual immunization planning reports from the National Health Commission and the National Disease Control Bureau, batch issuance data from the Central Inspection Institute, academic journals such as “China Vaccine and Immunization,” industry analysis reports from Guanyan Tianxia, and authoritative media reports such as Xinhua News Agency. Missing years are estimated using trend extrapolation and growth rate adjustment methods, with compound growth rates calculated based on known vaccination volumes in known years, corrected according to policy nodes, market penetration, and vaccine supply capacity, and verified through multi-source data cross-validation to ensure trend rationality.

#### 5.2.1 2016–2017: wait-and-see sentiment and slow initiation

In 2017, 1.455 million doses were administered throughout the year, but the market started slowly. This coincided with the 2017 trend, in which 94.59% of posts were neutral in public comments. The LDA theme shows that the discussion keywords focused on clinical diagnosis and treatment dimensions, while public understanding of HPV remained at the level of “disease treatment” rather than “disease prevention.”

#### 5.2.2 2018: suppression of vaccination behavior by negative sentiment

In 2018, the number of doses administered was 3.417 million, a year-on-year increase of 134.8%, but below market expectations. In 2018, the average emotional score plummeted to −0.776, Baidu Index shows that searches for “Is the HPV vaccine safe?” and “HPV vaccine side effects” increased by over 300% month-on-month, while searches for decision-making keywords like “HPV vaccine appointment” declined.

#### 5.2.3 2019–2020: rational return and accelerated vaccination

The number of doses administered increased from about 7 million doses in 2019 to 12.279 million doses in 2020, with a two-year compound annual growth rate of 89.7%. In 2020, sentiment data showed contradictory characteristics: negative comments accounted for the highest proportion (17.14%), but positive sentiment was more prevalent than negative sentiment (A2P ratio 0.21). Public discussion has shifted from “whether to get vaccinated” to “how to choose.”

#### 5.2.4 2021–2022: positive sentiment and explosive growth in vaccination

The vaccination volume soared from an estimated 25 million doses in 2021 to 62.48 million doses in 2022, a year-on-year increase of 149.9%. The average sentiment score in 2021–2022 turned positive for the first time (0.183). The rise in positive sentiment coincided with two major policies: the initiation of free vaccination pilots in multiple regions and the expanded age eligibility of the nine-valent HPV vaccine to 9–45 years. Sentiment spread rapidly through social media, forming a social climate. Meanwhile, problems such as “scalper appointments” also pushed up the negative comment proportion (13.39%).

#### 5.2.5 2023–2025: stabilized sentiment and normalized vaccination

The number of doses administered has rebounded from 58 million doses in 2023 to about 70 million doses in 2025, with growth clearly slowing and public sentiment shifting from “feverish” to “stable and rational.” LDA themes show that public awareness has expanded from “vaccination” to “full-cycle health management.”

## 6 Conclusion

This study analyzed 15,391 HPV vaccine-related texts collected from four major Chinese social media platforms—Zhihu, Weibo, Baidu Tieba, and Xiaohongshu—spanning from 2016 to 2026 using LDA topic modeling and lexicon-based sentiment analysis to investigate the evolution of public opinion on HPV vaccines in China. The study period was divided into three phases based on key policy milestones: Phase 1 (2016–2018), Phase 2 (2019–2024), and Phase 3 (2025–2026). For each phase, the LDA model extracted three core topics to trace the evolution of public concerns. The results show a clear thematic shift: from infection-focused discourse in Phase 1, to comprehensive prevention discussions in Phase 2, and finally to post-vaccination management concerns in Phase 3. Meanwhile, the sentiment analysis reveals a continuous improvement in public confidence: the proportion of positive posts rose from 21.3% in Phase 1 to 32.7% in Phase 2 and further to 38.5% in Phase 3, while the A2P ratio declined from 0.92 to 0.67 and then to 0.51. By further extracting specific time intervals driven by policy announcements and social hotspot events using Baidu Index, we analyzed user sentiment within these windows and found that positive sentiment experienced a relative decline in 2018 (45.5%, the lowest among all periods) following the Changchun Changsheng fake vaccine incident, recovering and reaching its highest level in 2021–2022 (58.1%), At the sam time free vaccination pilots and the 9-valent age expansion policy took effect.

This study explores the associations among policy events, social hotspots, public sentiment, and actual vaccination behavior within a unified analytical framework. Theoretically, it contributes to the computational social science literature by demonstrating how social media text mining can be integrated with behavioral data to track public health attitudes over long time spans. Practically, the findings provide empirical references for government agencies to anticipate public emotional responses to vaccine-related policies and to design targeted communication strategies. However, several limitations should be acknowledged. Due to platform anti-scraping mechanisms and the passage of time, earlier years suffer from more severe data incompleteness. The 2026 data, being a partial year, does not fully represent annual trends. The lexicon-based sentiment dictionary, while validated against human annotations, cannot accurately identify slang, sarcasm, abbreviations, and context-dependent expressions common in Chinese social media. Furthermore, vaccination volume data for several years are industry estimates rather than complete official statistics.

Future research can be advanced in several directions. Data sources should be expanded to include short-video platforms such as Douyin and Kuaishou, as well as multimodal content, to capture a more complete picture of public discourse. LLM can be employed to achieve fine-grained, context-aware sentiment analysis that better handles sarcasm and slang. Individual-level longitudinal surveys would complement aggregate social media data and help bridge the attitude–behavior gap. Finally, as the national immunization program continues to roll out, long-term policy effect assessments are needed, with particular attention to emerging issues such as male HPV vaccination and post-vaccination health management, providing actionable insights for future policy formulation.

## Data Availability

The original contributions presented in the study are included in the article/Supplementary material, further inquiries can be directed to the corresponding author/s.

## Supplementary material

Supplementary Table 1 LDA topic modeling results by phase.

Supplementary Table 2 HPV vaccine social media posts by platform (tieba).

Supplementary Table 3 HPV vaccine social media posts by platform (xiaohongshu).

Supplementary Table 4 HPV vaccine social media posts by platform (zhihu).

Supplementary Table 5 HPV vaccine social media posts by platform (weibo).

Supplementary Table 6 Sentiment analysis results.
